# Liquid Biopsy in Type 2 Diabetes Mellitus Management: Building Specific Biosignatures via Machine Learning

**DOI:** 10.3390/jcm11041045

**Published:** 2022-02-17

**Authors:** Makrina Karaglani, Maria Panagopoulou, Christina Cheimonidi, Ioannis Tsamardinos, Efstratios Maltezos, Nikolaos Papanas, Dimitrios Papazoglou, George Mastorakos, Ekaterini Chatzaki

**Affiliations:** 1 Laboratory of Pharmacology, Department of Medicine, Democritus University of Thrace, 68100 Alexandroupolis, Greece; mkaragla@med.duth.gr (M.K.); mpanagop@med.duth.gr (M.P.); chris_cheimonidi@imbb.forth.gr (C.C.); 2 JADBio Gnosis DA, Science and Technology Park of Crete, 71500 Heraklion, Greece; tsamard.it@gmail.com; 3 Diabetes Centre, 2nd Department of Internal Medicine, Democritus University of Thrace, University Hospital of Alexandroupolis, 68100 Alexandroupolis, Greece; emaltez@med.duth.gr (E.M.); papanasnikos@yahoo.gr (N.P.); dpapazog@med.duth.gr (D.P.); 4 Endocrine Unit, 2nd Department of Obstetrics and Gynecology, National and Kapodistrian University of Athens, “Aretaieion” University Hospital, 11528 Athens, Greece; mastorakg@gmail.com; 5 Institute of Agri-Food and Life Sciences, Hellenic Mediterranean University Research Centre, 71003 Heraklion, Greece

**Keywords:** type 2 diabetes, circulating cell free DNA, DNA methylation, machine learning

## Abstract

Background: The need for minimally invasive biomarkers for the early diagnosis of type 2 diabetes (T2DM) prior to the clinical onset and monitoring of β-pancreatic cell loss is emerging. Here, we focused on studying circulating cell-free DNA (ccfDNA) as a liquid biopsy biomaterial for accurate diagnosis/monitoring of T2DM. Methods: ccfDNA levels were directly quantified in sera from 96 T2DM patients and 71 healthy individuals via fluorometry, and then fragment DNA size profiling was performed by capillary electrophoresis. Following this, ccfDNA methylation levels of five β-cell-related genes were measured via qPCR. Data were analyzed by automated machine learning to build classifying predictive models. Results: ccfDNA levels were found to be similar between groups but indicative of apoptosis in T2DM. *INS* (Insulin), *IAPP* (Islet Amyloid Polypeptide-Amylin), *GCK* (Glucokinase), and *KCNJ11* (Potassium Inwardly Rectifying Channel Subfamily J member 11) levels differed significantly between groups. AutoML analysis delivered biosignatures including *GCK*, *IAPP* and *KCNJ11* methylation, with the highest ever reported discriminating performance of T2DM from healthy individuals (AUC 0.927). Conclusions: Our data unravel the value of ccfDNA as a minimally invasive biomaterial carrying important clinical information for T2DM. Upon prospective clinical evaluation, the built biosignature can be disruptive for T2DM clinical management.

## 1. Introduction

Just under half a billion people are living with diabetes worldwide, and the number is projected to increase by 25% in 2030 and 51% in 2045 [1]. Diabetes is a serious cause of blindness, kidney failure, stroke and amputations. Type 2 Diabetes mellitus (T2DM), the most common type of diabetes, is characterized by inadequate beta-pancreatic cell (β-cell) function, insulin insensitivity and chronic inflammation, all of which progressively lead to impaired glucose homeostasis [2]. In post-mortem specimens of T2DM patients, β-cell mass is reduced by 30–40% compared with specimens from non-diabetic subjects [3]. Increased β-cell apoptosis and reduced functional capacity of the remaining cells are important factors that contribute to the onset and the progression of the disease [4]. The inability to detect diabetes before the development of hyperglycemia limits our power for diagnosis prior to clinical onset and for earlier interventions to preserve significant β-cell mass. Established biomarkers for monitoring T2DM progression such as HbA1c levels are unable to consistently and non-invasively detect/monitor the ongoing β-cell destruction in islets. Thus, as the number of people with T2DM worldwide keeps on rising, there is an emerging need for the development of minimally invasive biomarkers for the early diagnosis of diabetes as well as the development of monitoring potential, both of which could lead to better therapeutic decisions.

Given the significant obstacle of directly accessing the pancreas for biopsy, special attention has been recently given to minimally invasive biomarkers for monitoring or diagnosing the disease early. Towards this direction, epigenetic information detected in circulating cell-free DNA (ccfDNA) is currently tested as a clinically valuable biomarker of β-cell death in Type 1 Diabetes Mellitus (T1DM) by several groups [5,6,7,8,9,10,11,12]. These biomarkers rely on the principles that necrotic or apoptotic cells or even viable cells release amounts of their DNA into the bloodstream and/or other biofluids that can be easily detected [13] and that each cell type has a unique and stable methylome that controls gene expression [14].

In the field of biomarker discovery, it becomes increasingly apparent that a single biochemical or epi/genetic feature is unlikely to bear the sensitivity/specificity required to disrupt clinical practice, and more effort is put into describing feature patterns or models. Machine Learning (ML) is the application of artificial intelligence on data analysis to build trained models [15]. ML has penetrated biomarker discovery in many diseases [16,17,18,19] and in diabetes [20,21]. Nowadays, Automate ML (AutoML) tools have become available. They promise to democratize data analysis to non-experts, improve the replicability of the statistical analysis, and shield against common methodological analysis pitfalls such as overfitting [22]. AutoML had been used for the prediction and diagnosis of diseases such as Alzheimer’s disease [23], lung and breast cancer [24,25,26], and suicide prediction amongst depressive patients [27].

**Figure 1:**
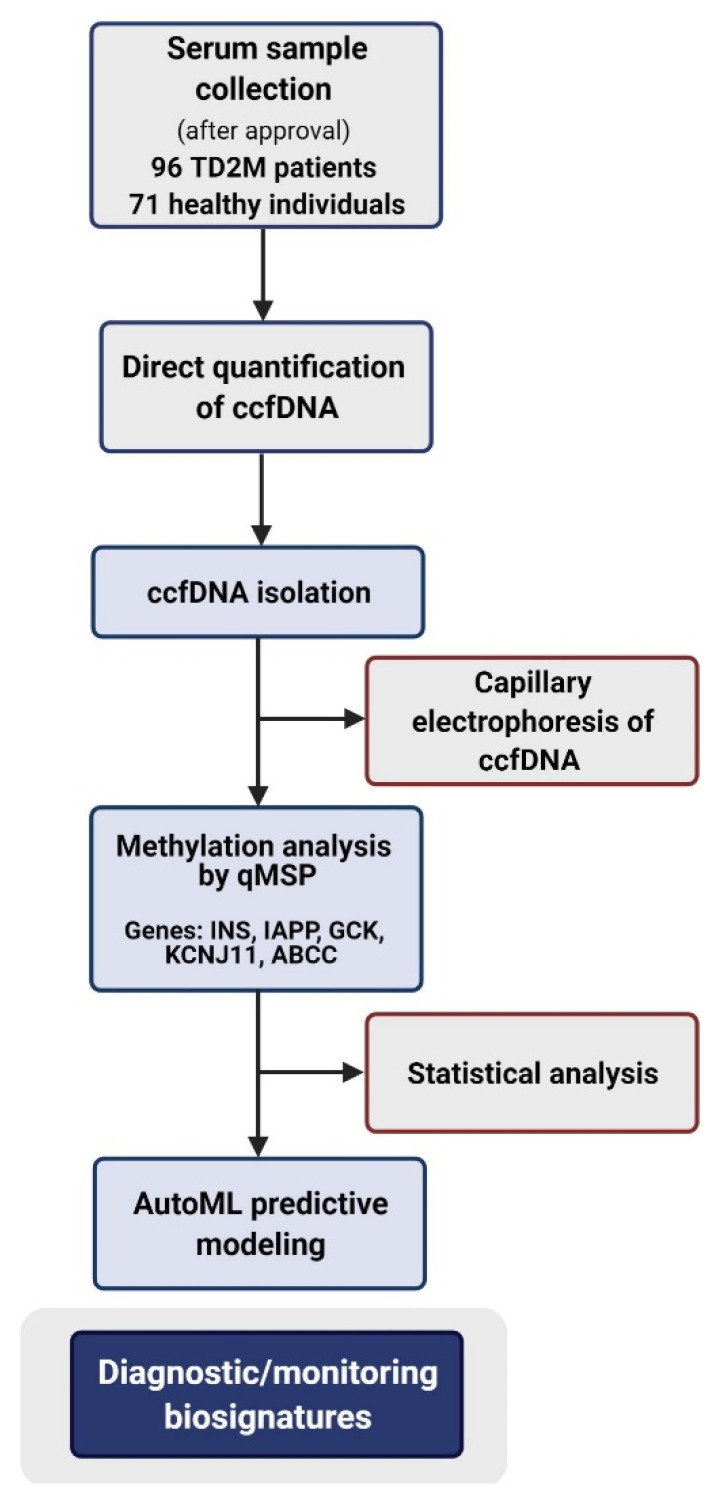
Workflow of our study. Abbreviations: TD2M: Type 2 diabetes mellitus, ccfDNA: circulating cell free DNA, qMSP: quantitative Methylation Specific PCR, AutoML: Automated Machine Learning.

In the present study, we focused on ccfDNA as a liquid biopsy biomaterial in T2DM, its characteristics and potential for clinical use. We first quantified ccfDNA levels of T2DM patients and healthy volunteers directly in serum, and we studied its fragment size distribution in order to evaluate its cellular release mechanism. We then evaluated the methylation profile of a panel of β-cell-related specific genes—*INS* (Insulin), *IAPP* (Islet Amyloid Polypeptide-Amylin), *GCK* (Glucokinase), *KCNJ11* (Potassium Inwardly Rectifying Channel Subfamily J member 11), and *ABCC8* (ATP Binding Cassette Subfamily C member 8)—via quantitative SYBR Green-based methylation-specific PCR. We selected this panel of β-cell-related specific genes based on existing literature proposing them as promising biomarkers for β-cell death (*INS*, *IAPP* and *GCK*) [28], as well as based on their implication for β-cell physiology (*INS*, *IAPP*, *GCK*, *KCNJ11* and *ABCC8*) [5,6,7,8,9,10,11,12,29] and potential response to antidiabetic therapy [30]. Most importantly, ad-hoc AutoML technology was applied on our ccfDNA experimental parameters in combination with patient demographical data (i.e., age, gender, BMI and smoking) to build accurate diagnostic/monitoring predictive biosignatures of clinical value for personalized diabetes management. Our study’s workflow is presented in Figure 1.

## 2. Results

### 2.1. ccfDNA Levels in T2DM Patients and Healthy Volunteers

**Figure 2:**
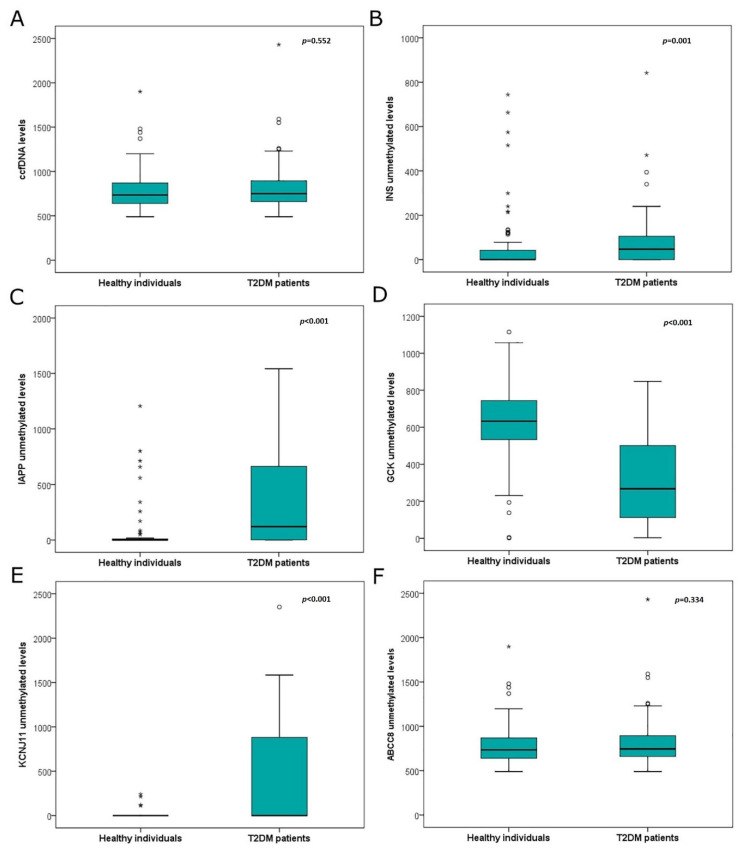
(**A**) Box plot of serum ccfDNA levels between healthy individuals (*n* = 71) and T2DM patients (*n* = 96) (*p* = 0.552). (**B**) Box plot of *INS* unmethylated alleles between healthy individuals and T2DM patients (*p* = 0.001). (**C**) Box plot of *IAPP* unmethylated alleles between healthy individuals and T2DM patients (*p* < 0.001). (**D**). Box plot of *GCK* unmethylated alleles between healthy individuals and T2DM patients (*p* < 0.001). (**E**) Box plot of *KCNJ11* unmethylated alleles between healthy individuals and T2DM patients (*p* < 0.001). (**F**) Box plot of *ABCC8* unmethylated alleles between healthy individuals and T2DM patients (*p* = 0.534). Small circles (°) correspond to “outlier” values and stars (*) to the “extreme” values of the dataset. Abbreviations: T2DM: Type 2 diabetes mellitus, ccfDNA: circulating cell free DNA.

Total ccfDNA levels were directly quantified in sera from T2DM patients and healthy individuals (control group). Interestingly, ccfDNA levels did not differ between healthy individuals and diabetes patients (700(400–1590) ng/mL vs. 820(490–2430) ng/mL, respectively, *p* = 0.552) (Figure 2). Then, ccfDNA levels were compared within the group of diabetes patients in relation to different clinical characteristics. T2DM patients with more than 14 years of diabetes showed significantly higher ccfDNA levels than those with less years (*p* = 0.010). Other clinical parameters, such as gender, smoking, Body Mass Index (BMI), and insulin or oral antidiabetic treatment, showed no correlations to ccfDNA levels.

### 2.2. ccfDNA Fragment Size Analysis in T2DM Patients and Healthy Volunteers

**Figure 3:**
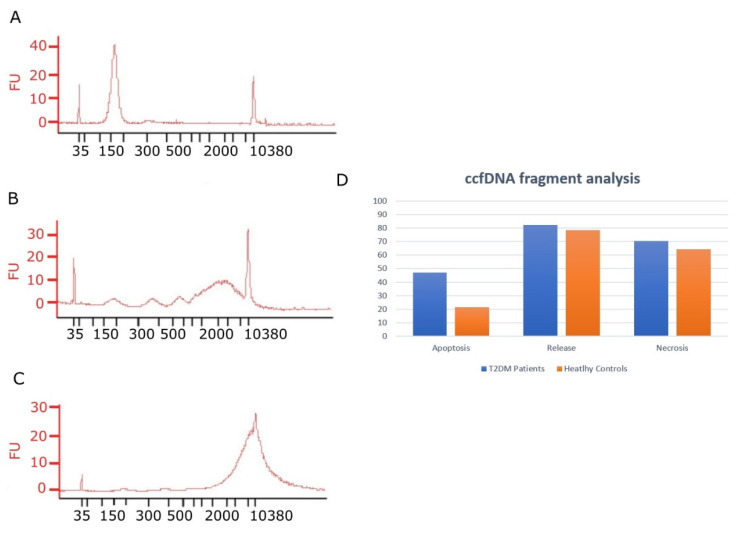
Representative capillary electropherograms showing DNA fragment size distribution in ccfDNA isolated from sera of two T2DM patients and one healthy control. (**A**) A T2DM patient ccfDNA sample showing a peak at ~160 bp indicative of apoptosis. (**B**) Another T2DM patient ccfDNA sample showing multiple peaks at ~160 bp, ~300 bp and ~500 bp indicative of apoptosis and an additional wide peak at ~2000–3000 bp indicative of active release. (**C**). A healthy volunteer ccfDNA sample with a wide peak at ~2000–3000 bp indicative of active release. Peaks at 35 bp and 10,380 bp in all electropherograms represent high and low ladders, respectively. (**D**) Distribution of ccfDNA fragment analysis in the patient’s group and the healthy volunteer’s group, respectively. Abbreviations: T2DM: Type 2 diabetes mellitus, ccfDNA: circulating cell free DNA, bp: base pairs.

DNA fragment size analysis was performed in isolated ccfDNA samples in order to reveal information about the cellular process of its release in the tissue of origin. It has been reported that samples containing DNA fragments of ~160 bp and multiples indicate release during apoptosis, whereas larger DNA fragments of ~2000–3000 bp are indicative of active release and above 10,000 bp are indicative of necrosis [31,32]. In the patients’ group, peaks of~160 bp (and multiples ×160 bp) were detected more often (47% of samples) than in healthy controls (21% of samples), indicating greater incidence of apoptosis (although the difference between percentages was not statistically significant). Peaks of larger DNA fragments of~2000–3000 bp were observed in both groups. Notably, the ~2000 bp fragments appeared more often in T2DM patients with higher BMI, considered a continuous variable (*p* = 0.037). In Figure 3, representative electropherograms from two T2DM patients and one healthy individual are presented, showing a different pattern.

### 2.3. Methylation Analysis of β-Cell-Specific Genes

Developed qMSP assays were validated in terms of analytical specificity, sensitivity, reproducibility, linearity and efficiency. The analytical specificity of all our developed assays was found to be 0.5% in the detection of methylated DNA molecules in a background of unmethylated DNA and 0.5% in the detection of unmethylated DNA molecules in a background of methylated DNA (specificity curves are presented in Supplementary Figure S1). The analytical sensitivity was found to be up to 0.1 ng for both methylated and unmethylated DNA molecules (sensitivity curves are shown in Supplementary Figure S1). The efficiency of all our developed assays ranged between 93 and 97%. Furthermore, high within—and between—sample reproducibility was also observed for all assays (CV ranging from 0.5% to 1.2%).

**Figure 4:**
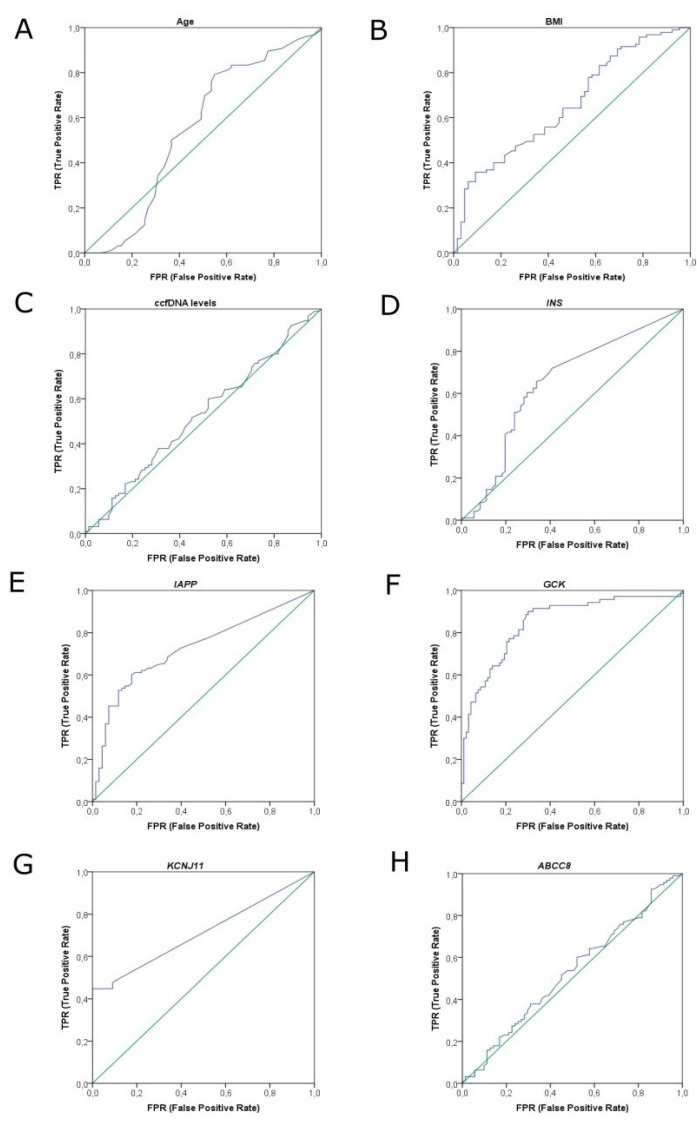
ROC curve analysis results. (**A**) ROC curve of age reaching an AUC of 0.566 (95% CI 0.468–0.664). (**B**) ROC curve of BMI reaching an AUC of 0.658 (95% CI 0.573–0.743). (**C**) ROC curve of ccfDNA levels reaching an AUC of 0.527 (95% CI 0.438–0.616). (**D**) ROC curve of *INS* gene reaching an AUC of 0.650 (95% CI 0.562–0.737). (**E**) ROC curve of *IAPP* gene reaching an AUC of 0.727 (95% CI 0.649–0.805). (**F**) ROC curve of *GCK* gene reaching an AUC of 0.848 (95% CI 0.787–0.910). (**G**) ROC curve of *KCNJ11* gene reaching an AUC of 0.712 (95% CI 0.619–0.806). (**H**) ROC curve of *ABCC8* gene reaching an AUC of 0.528 (95% CI 0.439–0.617). Abbreviations: ROC curve: receiver operating characteristic curve, BMI: Body Mass Index, AUC: area under the curve, CI: confidence interval, ccfDNA: circulating cell free DNA.

*INS*, *IAPP*, *GCK* and *KCNJ11* methylation differed significantly between T2DM patients and healthy individuals (*p* = 0.001, *p* < 0.001, *p* < 0.001 and *p* < 0.001, respectively), while *ABCC8* methylation did not differ between groups (*p* = 0.534) (Figure 2). Receiver operating characteristic (ROC) curve analysis showed that *GCK* methylation could provide high discrimination between T2DM patients and healthy individuals (AUC 0.848 (95% CI 0.787–0.910)) (Figure 4), while *IAPP* and *KCNJ11* methylation could offer lower discrimination between groups (AUC 0.727 (95% CI 0.649–0.805) and AUC 0.712 (95% CI 0.619–0.806), respectively) (Figure 4). *INS* methylation showed poor discrimination capacity between patients and controls (AUC 0.650 (95% CI 0.562–0.737)) (Figure 4).

Methylation profiles of T2DM patients with complications were similar in all studied genes to those of patients without complications. Moreover, the duration of diabetes (less or more than 15 years of disease) and the levels of HbA1c (less or more than 8%) did not correlate with gene methylation. Analysis of methylation with respect to the other clinicopathological characteristics, such as gender, smoking, Body Mass Index (BMI), and insulin or oral antidiabetic treatment, did not reveal significant correlations.

### 2.4. AutoML Predictive Analysis

Our data were further analyzed by ML techniques in order to produce diagnostic/monitoring biosignatures of clinical value, combining the novel liquid biopsy-based methylation data emerged by our study and the clinical and demographical data of the study’s groups. The JADBio automated machine learning (AutoML) platform employed for this analysis automatically performs and compares all standard, best practices and advanced ML techniques, and it produces upon feature selection the optimal best-performing model along with the most interpretable one.

**Figure 5:**
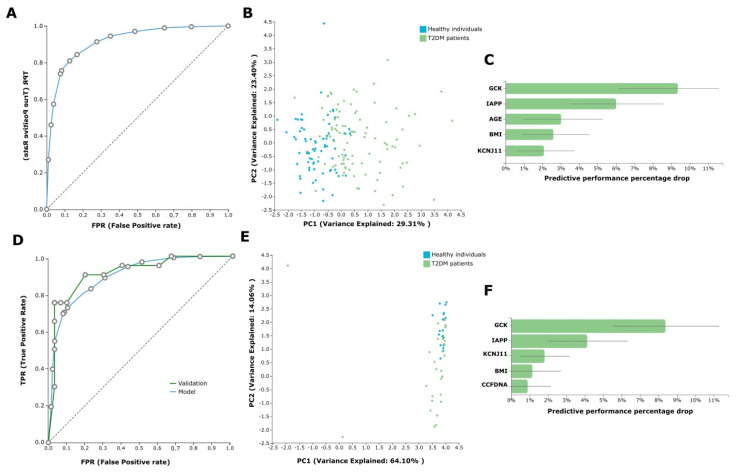
Predictive modelling results. (**A**) ROC curve of whole dataset reaching an AUC of 0.927 (95% CI 0.874–0.967). (**B**) Supervised Principal Component Analysis (PCA) plot of whole dataset depicting discrimination between T2DM patients and healthy individuals. (**C**) Feature importance plot of the features of the best-performing model for the whole dataset. Feature importance is defined as the percentage drop in predictive performance when the feature is removed from the model. (**D**) ROC curve of train sub-dataset (blue line) reaching an AUC of 0.898 (95% CI 0.845–0.944) and test sub-dataset (green line) showing an AUC of 0.923 for the best-performing model. (**E**) PCA plot of test sub-dataset depicting discrimination between T2DM patients and healthy individuals. (**F**) Feature Importance plot of the features of the best-performing model in the train/test 70/30 split sub-datasets. Abbreviations: ROC curve: receiver operating characteristic curve, AUC: area under the curve, CI: confidence interval, T2DM: type 2 diabetes mellitus.

In our AutoML analysis, the task was to predict T2DM versus health from the available ccfDNA parameters and the demographical patient data (age, gender, BMI, etc.). We first analyzed the whole dataset of the 96 T2DM patients on treatment and the 71 healthy individuals (control group). In this analysis, JADBio trained 3017 different machine learning pipelines (also called configurations), corresponding to different model types. Each one was employed many times during cross-validation (a repeated 10-fold CV without dropping), leading to fitting 90,510 model instances (https://app.jadbio.com/share/d59a08fb-e7ea-42e8-8eae-b225f512a38b, accessed on 13 January 2022). This classification analysis produced a best-performing five-feature biosignature via the Classification Random Forests algorithm that was able to discriminate between T2DM patients and healthy individuals with an AUC of 0.927 (95% CI 0.874–0.967) and an average precision of 0.951 (95% CI 0.914–0.980). Biosignature’s features included *GCK*, *IAPP* and *KCNJ11* methylation as well as age and BMI, and their contribution in the model’s performance defined as the percentage drop in predictive performance when the feature is removed from the model is shown in Figure 5C. The best-performing biosignature’s performance is presented in Figure 5A,B. The most interpretable five-feature biosignature was also built via a ridge logistic regression algorithm reaching an AUC of 0.915 (95% CI 0.868–0.957) and an average precision of 0.941 (95% CI 0.901–0.975). This biosignature included as features *GCK*, *IAPP* and *KCNJ11* methylation, smoking status and BMI.

Most importantly, the size of the dataset allowed for further model automated validation. The whole dataset was split randomly into training and test sub-datasets by a 70/30 ratio via JADBio. In this analysis, JADBio trained 3017 different machine learning pipelines, corresponding to different model types and fitted 150,850 model instances (https://app.jadbio.com/share/42c8c603-06d4-47e7-8276-97d4fa970d6c, accessed on 13 January 2022). The training data from 66 T2DM patients and 51 healthy individuals (control group) led to a similar but not identical best-performing five-feature biosignature via the Classification Random Forests algorithm, which was able to discriminate between patients and healthy individuals with an AUC of 0.898 (95% CI 0.845–0.944) and an average precision of 0.937 (95% CI 0.893–0.968) (Figure 5D,E). The biosignature’s features included *GCK*, *IAPP* and *KCNJ11* methylation as well as BMI and ccfDNA concentration, all but the last common to the original model from the whole dataset. Validating the model in the test sub-group data from 30 T2DM patients and 20 healthy individuals showed an AUC of 0.923 and an average precision of 0.945, verifying the model’s performance stability. The best-performing biosignature is presented in Figure 5D–F. The most interpretable five-feature biosignature was also built via the Ridge Logistic Regression algorithm reaching an AUC of 0.879 (95% CI 0.826–0.927) and an average precision of 0.921 (95% CI 0.881–0.957). This biosignature’s features included again *GCK*, *IAPP* and *KCNJ11* methylation, as well as BMI and ccfDNA concentration. In validation in the test dataset, model reached an increased AUC of 0.958 and an average precision of 0.972, again verifying no overfitting in the model construction. Supplementary Table S1 displays the algorithms and tuning hyper-parameter values that JADBio’s AI decided to try in the analysis of the splitted training dataset.

## 3. Discussion

ccfDNA is released into the blood, urine, and other biological fluids after cell apoptosis/necrosis or by active release from living cells [13]. Currently, ccfDNA-based biomarkers have emerged as promising minimally invasive options for the early diagnosis and monitoring of T1DM in several studies [5,8,11]. According to El Tarhouny et al., ccfDNA levels were significantly elevated in T2DM patients with or without complications compared to healthy individuals, indicating that ccfDNA can also be of significant clinical value in T2DM personalized management [33]. To expand knowledge on ccfDNA in T2DM with potentially valuable clinical applications, we investigated the levels, the fragment size distribution as well as the methylation profile of ccfDNA in a cohort of T2DM patients and healthy individuals with the ultimate goal of the production of diagnostic/monitoring biosignatures. To the best of our knowledge, this is the first study to evaluate such ccfDNA-based experimental parameters in a multi-level approach in diabetes.

By directly measuring ccfDNA in sera via fluorometry, our data revealed that ccfDNA levels were similar between T2DM patients and healthy individuals. This discrepancy with the data of El Tarhouny et al. [33] can possibly be attributed to different quantification methods and patient classification criteria. Thus, in our study, ccfDNA levels were quantified via fluorometry, counting both nuclear and mitochondrial fractions of free/naked ccfDNA (unbounded DNA). On the other hand, the quantification of extracted ccfDNA by PCR for the *GAPDH* gene that was used by El Tarhouny et al. measures only the nuclear fraction of extracted ccfDNA and both the unbounded and bounded DNA in protein complexes.

Plasma ccfDNA is a popular specimen for liquid biopsy approaches, although in several previous studies on beta-cell death serum samples were used for ccfDNA isolation, so we decided to keep up with these approaches in order to have comparable results [5,8,9]. In any case, our pilot experiments not included in the manuscript showed no significant differences in the yield and quality of ccfDNA isolated between plasma and serum blood fractions. Serum volumes as low as those used here have not been highly reproducible in past assessments of similar assays; indeed, the cancer field where these assays are most highly advanced are in many cases moving toward sample volumes an order of magnitude larger than those used here. Our results from direct and indirect quantifications demonstrate that used sample volume was sufficient for the downstream analysis, as previously demonstrated by our group and others [9,25]. In addition, taking into consideration the short half-life of ccfDNA fragments and in order to avoid significant loss, isolation protocol was initiated within two hours of collection.

Several suggested cellular processes are responsible for ccfDNA release into biological fluids and the size of the DNA fragment content is informative for each one of them. Apoptosis is characterized by ~160 bp and multiple fragments, necrosis delivers fragments above ~10,000 bp, while active release delivers ~2000–3000 bp fragments [31,32]. We have recently shown that in breast cancer patient samples, DNA fragments of ~160 bp and multiples and those of ~10,000 bp appeared more often (in 60.0% and 53% of patients, respectively) than DNA fragments of ~2000 bp (in 37% of patients) [25]. In our previous study, using two cancer cell lines, we have shown that induction of apoptosis in vitro resulted in the release of these short ~160 bp and multiple fragments, confirming these data [34]. To reveal the mechanism(s) of ccfDNA release into the circulation in T2DM, fragment analysis by capillary electrophoresis showed mainly fragments of ~160 bp and multiples and that of ~2000 bp inT2DM patients, indicating greater incidence of apoptosis, although not reaching statistical significance. This could be attributed to the ongoing apoptosis of remaining functional insulin-producing β-cells in accordance with the fact that β-cell destruction is an important etiological factor in the development and progression of T2DM [35]. Moreover, in obese T2DM patients, peaks of ~2000 bp fragments were observed more often than in non-obese patients, possibly suggesting that obesity-induced DNA release from adipocytes is via active release.

In order to examine the tissue origin of ccfDNA in T2DM, we investigated the methylation status of five β-cell-specific genes based on previous literature findings that propose them as promising biomarkers for β-cell death (*INS*, *IAPP* and *GCK*), as well as their implication on β-cell physiology (*INS*, *IAPP*, *GCK*, *KCNJ11* and *ABCC8*). As methylation is a tissue-specific event, genes expressed exclusively in β-cells are unmethylated only in this cell type, and this status is expected to be detectable also in ccfDNA released by them. It has been previously shown that *INS*, *IAPP* and *GCK* are found to be unmethylated in serum ccfDNA of T1DM patients in relation to healthy individuals [5,6,8,9,10,11]. We have also previously studied the methylation levels of *KCNJ11* and *ABCC8* in another cohort of T2DM patients regarding their response to antidiabetic therapy, and we were interested in investigating further their use as biomarker of T2DM [30]. Here, we focused on T2DM and the methylation status of *INS*, *IAPP*, *GCK* and *KCNJ11*, and *ABCC8* is investigated for the first time in serum ccfDNA of T2DM patients. All but the *ABCC8* gene showed statistically significant differential methylation levels between patients and controls. Higher percentages of unmethylated alleles show the beta-pancreatic origin of ccfDNA in the blood of T2DM patients. It can be concluded that while on an apoptotic cell death pathway, β-cells release ccfDNA and enrich the physiological pool of free nucleic acids in the circulation.

To further exploit our ccfDNA experimental observations in order to build classifying biosignatures of higher diagnostic/monitoring performance, we performed classification analysis by employing an innovative AutoML approach. We used JADBio, an AutoML platform that uses state-of-the-art statistical and machine learning methods [22]. Another innovative element of our approach was combining all ccfDNA experimental parameters (including levels and gene methylation) with clinical and demographical patient characteristics in one analysis executing feature selection and therefore eliminating redundancy. AutoML analysis in the dataset as a whole delivered a best-performing five-feature biosignature via the Classification Random Forests algorithm, including *GCK*, *IAPP* and *KCNJ11* methylation and age and BMI, showing very high performance in discriminating T2DM patients from healthy individuals (AUC 0.927). Similarly, the AutoML of split train/validation data led to a best-performing five-feature biosignature via the Classification Random Forests algorithm with the same but one new feature, i.e., *GCK*, *IAPP* and *KCNJ11* methylation and BMI and ccfDNA concentration, showing nearly as high discrimination capacity between groups (AUC 0.898). This was expected, as models built in smaller datasets demonstrate compromised performance. Most importantly, the validation of this biosignature in the independent validation subgroup showed an AUC of 0.923, verifying the model’s performance stability and the absence of overfitting, which confirms what claimed to be achieved by the AutoML tool used. In both models, *GCK* and *IAPP* methylation are found to be the major features contributing to the performance, highlighting their value as biomarkers in diabetes.

To fully assess clinical relevance, we next plan to validate these biosignatures in independent, blinded validation cohorts of T2DM patients, obese and prediabetic individuals from a different clinical setting (cross-dataset analysis). We should note, however, that JADBio implements internal cross-validation with the bootstrap corrected cross-validation (BBC-CV) algorithm [36,37], shown to substitute external validation. The bootstrapping technique performs a correction to the estimation of out-of-sample performance of the final model. The correction (adjustment) is required because JADBio tries thousands of machine learning pipelines to identify the best one that produces the optimal, final model. The correction is conceptually similar to the Bonferroni adjustment required for multiple hypotheses testing due to performing multiple tests. Intuitively, the selection process, which selects the best out of numerous pipelines is bootstrapped. Internally, JADBio holds out a subset of the training data to test the generalization performance (out-of-sample) of the produced model, effectively simulating the presence of an external dataset. In fact, JADBio performs this training and test procedure numerous times to reduce the variance of the estimation. Specifically for small sample sizes, it employs a stratified, K-fold, repeated cross-validation protocol that exhibits small estimation variance. In addition, the final estimate is corrected for the fact that numerous machine learning pipelines have been tried (a form of the “winner’s curse”) using the recently developed bootstrap corrected cross-validation (BBC-CV) algorithm. This technique has been shown to produce conservative estimates of performance in massive evaluation experiments with general types of data [22]. In addition, the accurate estimation of AUC by JADBio in small sample settings has been tested in numerous studies. Indeed, we and others have previously shown in multimodal datasets that AUC estimations did not drop upon external validation, showing no over-estimations [25,26,27,38,39,40]. This body of work proves that JADBio estimates can be trusted and there is no need to have a separate hold-out dataset to statistically validate the results, a feature of particular importance for maximal extrapolation of precious biomedical datasets. In the case presented here, the model built in the dataset as a whole had marginally better performance that the one built in 70/30 train/test. Still, as we ourselves would like to promote trustworthiness of this ML approach, we plan to re-optimize and externally evaluate the biosignatures built in real-world prospective setting.

A relatively small sample size is a limitation in this study. We [22] and others [40] argue that sample size is one of many important design elements contributing to the successful implementation in biomarker discovery. Machine learning, quickly penetrating the field, is there in order to overcome such limitations, aiding robust, optimized and maximal data extrapolation from small cohorts. As such, it is included in the recommended generic steps for robust model building and evaluation. The novel biosignatures presented in our study, based on qMSP rather than whole-genome or digital PCR methodologies, have a great translatability to cost-effective assays, which can be implemented in any standard molecular diagnostic laboratory. They are, therefore, readily available to offer feasible liquid biopsy-based assays for predicting or diagnosing early diabetes as well as monitoring β-cell death upon prospective clinical validation.

Previous studies leveraged demographical, clinical and laboratory data, such as age, BMI, glycated hemoglobin (HbA1c), hypertension, smoking and glucose, and employed ML tools for predicting risk of diabetes with promising results [20,21,41]. The predictive abilities of the models built, however, (AUC ranging from 0.826 to 0.872) were inferior to those presented here (AUC reaching 0.927) incorporating for the first time parameters of a liquid biopsy biomaterial such as ccfDNA with demographical data.

Other approaches focusing on liquid biopsy biomarkers for the management of diabetes have examined methylation of *INS*, *IAPP* and *GCK* either as single parameters [5,8,9,12] or as combined parameters, i.e., *INS* and *CHTOP* in a duplex assay [42]. Previous reports showed that *GCK* methylation is a more suitable marker than *INS* methylation for the detection of β-cell death in T1DM and therefore can present an early diabetes biomarker [9]. Furthermore, the recent study of Arosemena et al. reported that none of the four groups (lean controls with normal glucose tolerance; overweight/obese with normal glucose tolerance; impaired glucose tolerance; and T2DM patients) showed statistically significant differences in *INS* methylation compared to the healthy controls [43]. These findings are in agreement with our observations in T2DM, where *INS* methylation showed a low capacity to discriminate T2DM, and *GCK* methylation revealed the best capacity, highlighting its potential clinical value as a minimally invasive biomarker for T2DM personalized management. Our model incorporates this biomarker with the maximal feature importance (*GCK*) in the model’s performance, which, however, is dramatically increased by including other selected pancreatic gene methylation biomarkers and other parameters. These corroborated findings considered together, support the value of the herein model in the early detection of diabetes onset.

## 4. Materials and Methods

### 4.1. Study Groups and Serum Sampling

Our study’ s groups consisted of 96 T2DM patients on treatment and 71 healthy individuals (control group) of similar age without a history of diabetes. All samples were of Caucasian origin. T2DM was diagnosed according to the ADA guidelines [44]. All T2DM patients were on oral antidiabetic therapy of metformin, and 5 of them also received sulfonylureas treatment. Almost half of patients (*n* = 42) were also on insulin therapy. Demographic and clinical data of study groups are shown in Supplementary Table S2. Inclusion criteria of the study included age between 25 and 75 years old and ability to give informed consent. Exclusion criteria of all participants included the presence of a (another) chronic disease, underlying malignancies and systemic lupus erythematosus. Serum samples were obtained within 2 h of blood sampling through centrifugation at 3000× *g* for 10 min. An additional high-speed centrifugation step at 14,000× *g* for 10 min was performed to remove any cellular debris and contaminants, such as gDNA from damaged blood cells. Serum samples were stored at −80 °C until further use.

### 4.2. Direct Quantification of ccfDNA

The direct quantification of free unbounded/naked ccfDNA in 20 μL of serum was performed utilizing the Quant-iT dsDNA High-Sensitivity Assay Kit in Qubit 3.0 Fluorometer (Invitrogen, Karlsruhe, Germany) according to manufacturer specifications. A standard curve was generated using provided standards (0 and 10 ng/μL).

### 4.3. ccfDNA Isolation

All ccfDNA (naked, bound in nucleosomes or proteins, or internalized in vesicles) was extracted from 600 μL of serum using the QIAamp Blood Mini kit (Qiagen, Hilden, Germany) in a final elution volume of 30 μL, as specified by the manufacturer. ccfDNA isolation was performed manually in batches of 10 samples and no carrier DNA was used. Specifically, 20 μL Protease and 600 μL of each sample were added to a microcentrifuge tube, and then 200 μL Buffer AL was added to each sample. After lysis, samples were incubated for 10 min at 56 °C, and 200 μL 100% ethanol were added to each sample. Following this, the mixtures were applied to a QIAamp Mini spin column and two steps of wash and centrifuging at 6000× *g* were followed. Finally, 30 μL Buffer AE was added, and, through a new centrifugation step, serum ccfDNA was eluted.

The extracted ccfDNA was stored at −20 °C until further use. Then, 4 μL of extracted ccfDNA was subjected to quantitative PCR (qPCR) for the nuclear *GAPDH* gene to determine isolation efficiency in terms of ccfDNA quantity and quality following MIQE guidelines [45]. Primer sequences are shown in Supplementary Table S2.

### 4.4. Capillary Electrophoresis of Extracted ccfDNA

The High-Sensitivity DNA Kit on 2100 Bioanalyzer (Agilent Technologies, Santa Clara, CA, USA), an automated on-chip electrophoresis system, was used for the fragment size evaluation of 2 μL of extracted ccfDNA, following the manufacturer’s instructions.

### 4.5. Methylation Analysis

A total of 20 μL of extracted ccfDNA was treated with sodium bisulfite (SB) using the EZ DNA Methylation-Gold kit (ZYMO Research Co., Irvine, CA, USA) in a final elution volume of 10 μL, following the manufacturer’s procedure. In each reaction, CpGenome Human methylated and non-methylated DNA controls (Merck Millipore, Darmstadt, Germany) were included as negative and positive control samples, respectively. The SB-treated ccfDNA was stored at −80 °C until further use.

A methylation-independent PCR assay for the β-actin gene (*ACTB*) was used in order to verify sufficient quality and quantity of SB-treated ccfDNA. Methylation levels of β-cell-related genes *INS*, *IAPP*, *GCK*, *KCNJ11* and *ABCC8* were analyzed using quantitative SYBR Green-based methylation-specific PCR assays. For all genes, primers specific for methylated (m) and unmethylated (u) alleles were either newly designed using the MethPrimer [46] program or were based on bibliography with some modifications. Primer sequences are provided in Supplementary Table S3. To set up robust qMSP assays, extensive optimization was performed. Specificity and cross-reactivity of primers were evaluated using unconverted DNA, SB-treated methylated DNA and non-methylated DNA controls. Analytical specificity of qMSP assays was evaluated by using mixes of SB-converted methylated and non-methylated DNA standards (100%, 50%, 10%, 5%, 0.5%, 0%). Analytical sensitivity of assays was evaluated using serial dilutions of SB-treated methylated and non-methylated DNA controls in H_2_O. The reproducibility (calculated as coefficients of variation, CVs), the efficiency and the linearity were also evaluated in order to complete the validation file of the established assays. All samples were run in duplicates. The percentage of methylation in a sample was estimated using following formula by Lu et al. [47]:

(1) $$1-\frac{1}{1+2^{\left(-\Delta \mathrm{Ct}\right)}}\times 100\%$$

where

ΔCt = Ct_unmeth_ − Ct_meth_ (2)

Following this, the percentage of methylation was multiplied by the concentration of total serum ccfDNA.

### 4.6. Statistics

Initially, Kolmogorov–Smirnov test was applied to check for normality in distribution of continuous data. Due to lack of normality in our data, non-parametric statistics were used for comparisons between groups (Kruskal–Wallis test or Mann–Whitney U test). The Spearman correlation coefficient was used as a measurement of correlation for continuous variables. The predictive power of the ccfDNA levels and β-cell-related genes methylation was tested using receiver operating characteristic (ROC) curve analysis and Area Under the Curve (AUC) metric. Continuous variables are expressed as median value (25th–75th percentile, interquartile range). Categorical variables are shown as absolute frequencies (percentage). In all tests performed, statistical significance was set at two-sided *p* value < 0.050. Standard statistical analysis was carried out with the SPSS version 21.0 statistical software package for Windows (IBM-SPSS Inc., New York, NY, USA).

### 4.7. AutoML Predictive Modelling with JADBio

The innovative AutoML technology JADBio, version 1.2.8 (https://www.jadbio.com/, accessed on 13 January 2022) [22], was applied to produce diagnostic/monitoring biosignatures/classifiers based on the ccfDNA parameters, methylation data and demographical information. Given a 2D matrix of data, JADBio automatically preprocesses data (Mean Imputation, Mode Imputation, Constant Removal, Standardization), performs feature selection by employing LASSO or Statistical Equivalent Signatures (SES) algorithms, tries several algorithms (Classification Random Forests, Support Vector Machines (SVM), Ridge Logistic Regression and Classification Decision Trees) and thousands of algorithmic configurations, and then selects the best-performing model, estimates the out-of-sample model’s performance after bootstrap correction and cross-validation, and provides several visualizations.

The training set is used to train multiple machine learning pipelines that include the steps of preprocessing, imputation, feature selection, and modeling. Internally and automatically, JADBio holds out a subset of the training data to test the generalization performance (out-of-sample) of the produced model, effectively simulating the presence of an external dataset. In fact, JADBio performs this training and test procedure numerous times to reduce the variance of the estimation. Specifically, for small sample sizes, it employees a stratified, K-fold, repeated cross-validation protocol BBC-CV algorithm [37] that exhibits small estimation variance and removes the estimation bias due to the fact that it was selected among many. The features selected in the winning pipeline are the ones included in the returned model. In addition, our dataset was randomly automatically split into train and validation sub-datasets by a 70/30 ratio via JADBio.

For our AutoML classification analysis, we used extensive model tuning effort, we chose the AUC metric for optimization of performance and we set classifier maximum size to five features. The predictive power of the biosignature was assessed using AUC and average precision (also known as area under the precision–recall curve) metrics.

## 5. Conclusions

The identification of markers that can predict future onset of type 2 diabetes is of great interest to the field; the ability to measure such markers consistently and uninvasively is critical. Our data unravel the value of ccfDNA as a liquid-biopsy biomaterial carrying important clinical information for minimally invasive T2DM diagnosis and monitoring. Overall, by adopting ad-hoc AutoML technology in our study, a highly potent predictive biosignature based on ccfDNA methylation parameters in combination with demographical data has emerged, readily available to be translated into a cost-effective laboratory test. Upon prospective clinical evaluation, our biosignature could aid the early diagnosis and monitoring of T2DM, meeting the need for a minimally invasive advancement in the direction of personalized diabetes management.

## 6. Patents

The contents of this manuscript were included in an EPO patent filing. Makrina Karaglani, Ioannis Tsamardinos and Ekaterini Chatzaki act as inventors.

## Data Availability

The datasets generated/analyzed during the current study are available from the corresponding author on reasonable request.

## Supplementary Materials

The following are available online at https://www.mdpi.com/article/10.3390/jcm11041045/s1, Figure S1: (a) Specificity of *ABCC8* qMSP assay for the methylated primers set: amplification curves of 100%, 50%, 10%, 5% and 0.5% SB-converted methylated DNA standards; (b) Sensitivity of *KCNJ11* qMSP assay for the unmethylated primers set: amplification curves of 10-fold serially diluted 100% SB-converted non-methylated DNA standard-efficiency 93%; (c) Sensitivity of *INS* qMSP assay for the unmethylated primers set: amplification curves of 10-fold serially diluted 100% SB-converted non-methylated DNA standard-efficiency 97%; (d) Specificity of *INS* qMSP assay for the methylated primers set: amplification curves of 100%, 50%, 10%, 5% and 0.5% SB-converted methylated DNA standards; (e) Sensitivity of *IAPP* qMSP assay for the unmethylated primers set: amplification curves of 10-fold serially diluted 100% SB-converted non-methylated DNA standard-efficiency 96%; (f) Specificity of *GCK* qMSP assay for the unmethylated primers set: amplification curves of 100%, 50%, 10%, 5% and 0.5% SB-converted non-methylated DNA standards; Table S1: Demographic and clinical data of study groups; Table S2: Primer sequences, genomic locations, and related references (where relevant) used for qMSP assays.
