# Supplementary material for: Liquid Biopsy in Type 2 Diabetes Mellitus Management: Building Specific Biosignatures via Machine Learning
Source: J Clin Med. 2022 Feb 17;11(4):1045. doi: 10.3390/jcm11041045 (PMC8876363; doi:10.3390/jcm11041045)
Supplement: Supplementary file 1 [file jcm-11-01045-s001.zip › jcm-1582359-supplementary.pdf]

Journal of
Clinical Medicine

MDPI

Supplementary Material

Supplementary Table S1. Demographic and clinical data of study groups.

ALGORITHM TYPE	ALGORITHM	HYPER-PARAMETER	SET OF VALUES
**PREPROCESSING**	Mode imputation		
	Mean imputation		
	Contant Removal		
	Standardization		
**FEATURE SELECTION**	Test-Budgeted Statistically Equivalent Signature (SES)	alpha	0.1, 0.01, 0.05
		maxk	3, 2
	LASSO	penalties	1.25, 2.0, 0.0, 0.25, 1.0, 1.5, 0.5
**MODELING**	Linear Support Vector Machines	costs	0.1, 1.0, 0.001, 100.0, 10.0, 0.01
	Polynomial Support Vector Machines	gammas	1.0, 0.001, 0.1, 0.01, 10.0, 100.0
		costs	0.1, 1.0, 0.001, 100.0, 10.0, 0.01
		degrees	4, 2, 3
	RBF Support Vector Machines	gammas	1.0, 0.001, 0.1, 0.01, 10.0, 100.0
		costs	0.1, 1.0, 0.001, 100.0, 10.0, 0.01
	Logistic Regression	lambdas	1.0, 0.001, 0.01, 100.0, 10.0, 0.1, 1.0E-4
	Random Forests	min leaf sizes	1, 4, 3, 5, 2
		vars to split	0.816 sqrt (nvars), 1.0 sqrt (nvars), 1.291 sqrt (nvars),

			0.577 sqrt (nvars), 1.414 sqrt (nvars), 1.154 sqrt (nvars)
		splits to perform	1.0
		ntrees	1000, 100
	Decision Tree	min leaf sizes	2, 1, 4, 3, 5
		vars to split	nvars // 1.0
		splits to perform	1.0
		alphas	0.1, 0.05, 0.01

Supplementary Table S2. Demographic and clinical data of study groups.

	T2DM PATIENTS (N=96)	HEALTHY VOLUNTEERS (N=71)	P-VALUE
GENDER (MALE)	58 (60%)	50 (70%)	0.229
AGE (YEARS)	64 ±8	62 ±10	0.249
BMI (KG/M^2)	30.9 ±4.7	28.4 ±5.2	0.049
SMOKING	16 (17%)	25 (39%)	0.005
GLUCOSE (MG/DL)	138 ±37	85 ±13	0.475
C-PEPTIDE (NG/ML)	4.1 ±3.2	-	
HBA1C (%)	7.2 ±1.1	-	
DIABETES DURATION (YEARS)	14 ±9	-	
DIABETES COMPLICATION (PRESENCE)	25 (26%)	-	

**DIABETES THERAPY (YES)**	96 (100%)	**-**
**ORAL THERAPY (METFORMIN, ETC.)**	96 (100%)	**-**
**INSULIN**	42 (44%)	-

Supplementary Table S3. Primer sequences, genomic locations, and related references (where relevant) used for qMSP assays.

PRIMER NAME	PRIMER SEQUENCE (5′- 3′)	GENOMIC LOCATION (GRCH38)	REFERENCE
**GAPDHF**	CCC CAC ACA CAT GCA CTT ACG	12:6,535,875:1	[30]
**GAPDHR**	CCT AGT CCC AGG GCT TTG ATT	12:6,535,972:1	
**ACTBF**	TGG TGA TGG AGG AGG TTT AGT AAG T	7:5,558,705:-1	
**ACTBR**	AAC CAA TAA AAC CTA CTC CTC CC	7:5,558,838:-1	
**INSMETHF**	**CG**G AAA TTG TAG TTT TAG TTT TTA GTT ATT TGT C	11:2,159,776:-1	
**INSMETHR**	CCT AAA AAA CTA AAA ACT ACT AAA CCC C**CG**	11:2,159,922:-1	
**INSUNMETHF**	TGG AAA TTG TAG TTT TAG TTT TTA GTT ATT TGT T	11:2,159,776:-1	
**INSUNMETHR**	CCT AAA AAA CTA AAA ACT ACT AAA CCC CCA	11:2,159,922:-1	
**IAPPF**	TGT TAT TAG TTA TTA GGT GGA AAA G	12:21,378,228:1	[8]
**IAPPMETHR**	TAA AAA ATT TAC CAA A**CG** CTA **CG**	12:21,378,304:1	
**IAPPUNMETHR**	TAA AAA ATT TAC CAA ACA CTA CA	12:21,378,304:1	
**GCKMETHF**	AAT GT**C G**AG **CG**G **CG**T TTG AG	7:44,153,026:-1	[9]
**GCKUNMETHF**	AGG AAA TGT TGA GTG GTG TTT GAG T	7:44,153,022:-1	

**GCKR**	ATC CTC TCC CTT CTA TAA CCT AAA AAC AAC	7:44,153,136:-1
**KCNJ11METHF**	**CG**G GTT T**CG** GTT T**CG** TTC	11:17,389,140:1
**KCNJ11METHR**	CA**C G**AA **CG**A ACA AAC AAA **CG**	11:17,389,253:1
**KCNJ11UNMETHF**	TGG GTT TTG GTT TTG TTT GTT GT	11:17,389,140:1
**KCNJ11UNMETHR**	ACC CAC AAA CAA ACA AAC AAA CA	11:17,389,254:1
**ABCC8METHF**	TAG GAA GA**C G**TG **CG**G TAT TAC	11:17,476,660:1
**ABCC8METHR**	CTA **CG**A CAA **CG**A AAA CCA CT	11:17,476,743:1
**ABCC8UNMETHF**	GAG TAG GAA GAT GTG TGG TAT TAT	11:17,476,657:1
**ABCC8UNMETHR**	CTT CTA CAA CAA CAA AAA CCA CT	11:17,476,743:1

F: forward, R: reverse, METH: methylated, UNMETH: unmethylated

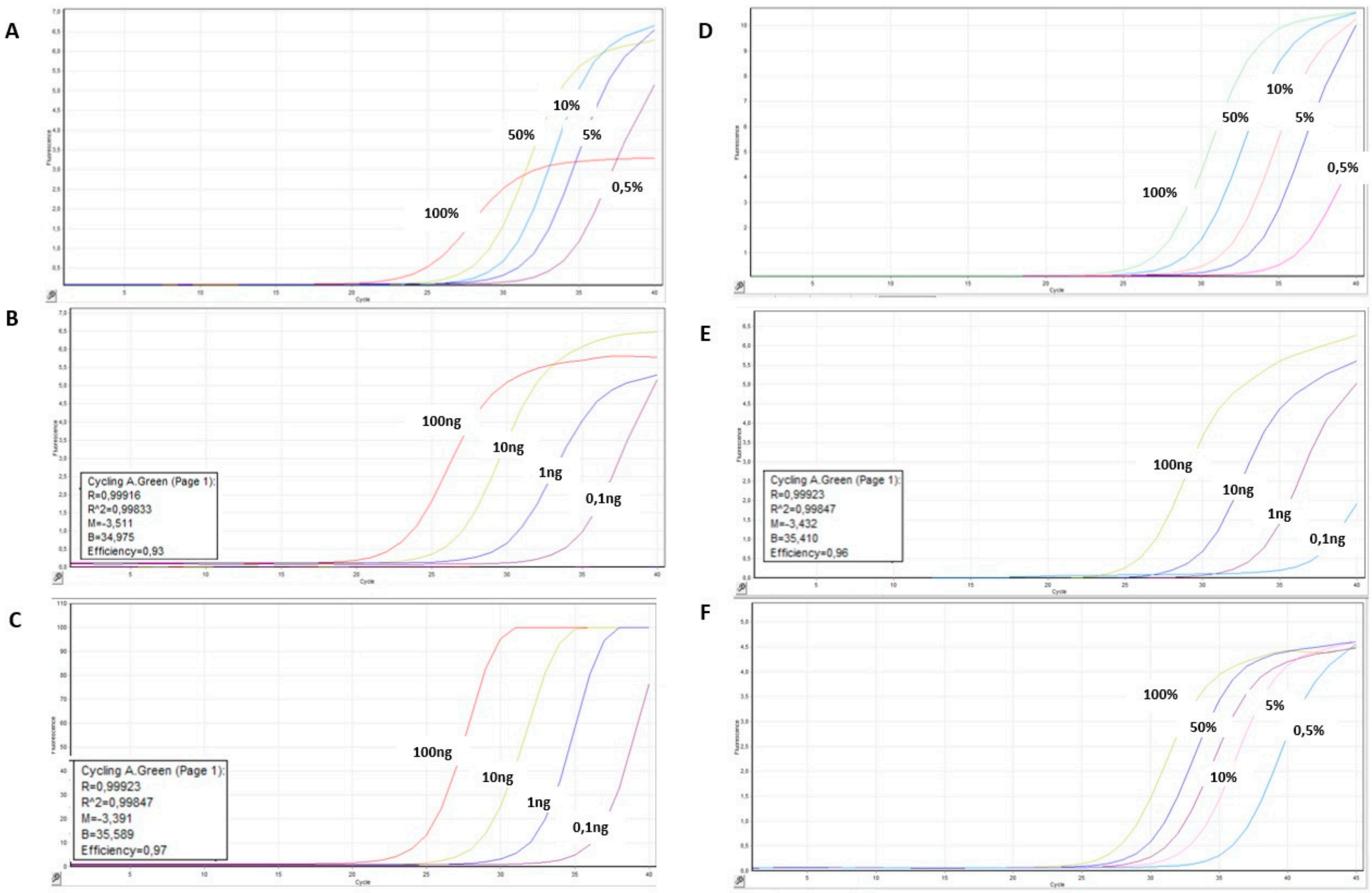

Supplementary Figure S1. (A) Specificity of *ABCC8* qMSP assay for the methylated primers set: amplification curves of 100%, 50%, 10%, 5% and 0.5% SB-converted methylated DNA standards. (B) Sensitivity of *KCNJ11* qMSP assay for the unmethylated primers set: amplification curves of 10-fold serially diluted 100% SB-

converted non-methylated DNA standard—efficiency 93%. (C) Sensitivity of *INS* qMSP assay for the unmethylated primers set: amplification curves of 10-fold serially diluted 100% SB-converted non-methylated DNA standard—efficiency 97%. (D) Specificity of *INS* qMSP assay for the methylated primers set: amplification curves of 100%, 50%, 10%, 5% and 0.5% SB-converted methylated DNA standards. (E) Sensitivity of *IAPP* qMSP assay for the unmethylated primers set: amplification curves of 10-fold serially diluted 100% SB-converted non-methylated DNA standard—efficiency 96%. (F) Specificity of *GCK* qMSP assay for the unmethylated primers set: amplification curves of 100%, 50%, 10%, 5% and 0.5% SB-converted non-methylated DNA standards.

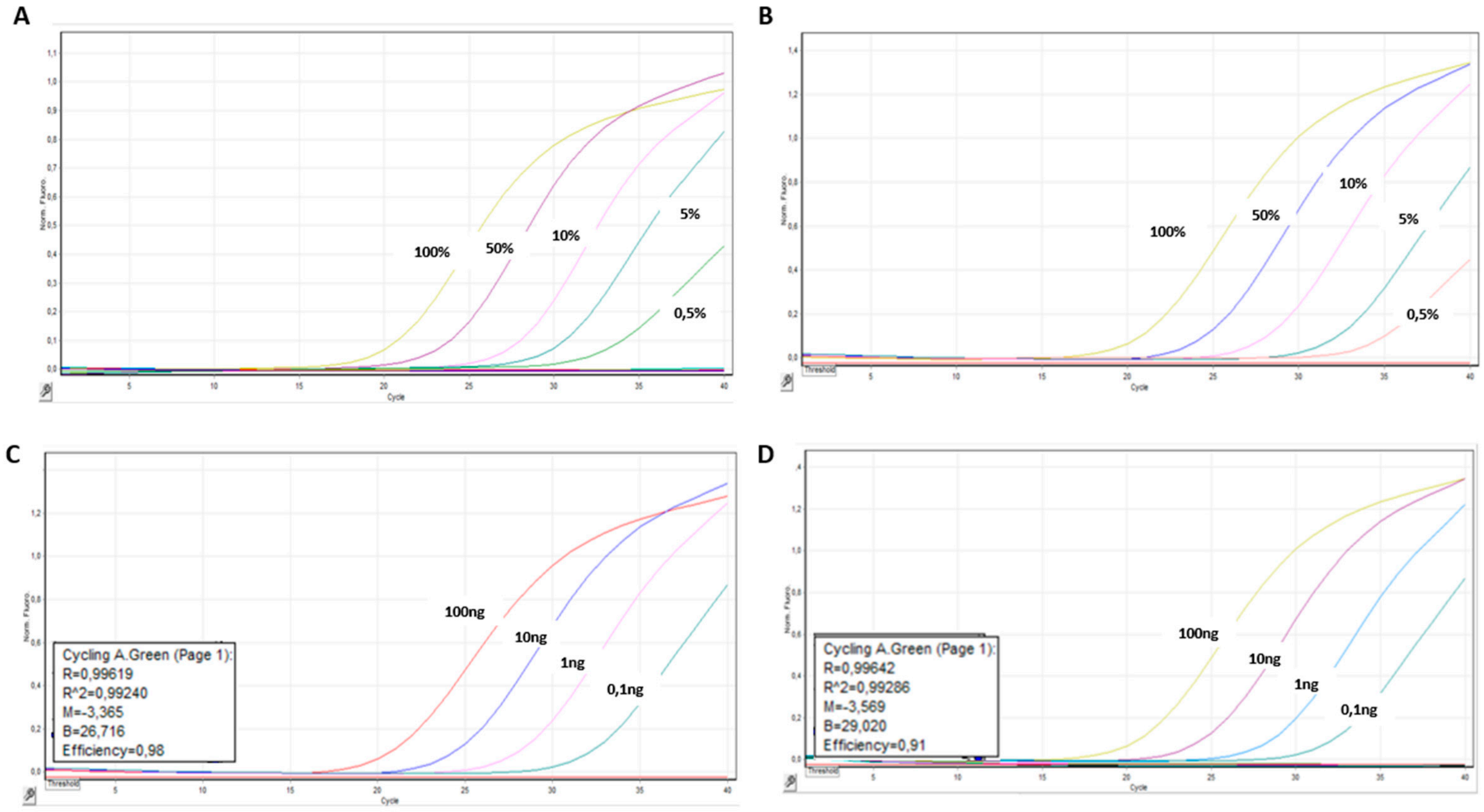

Supplementary Figure S2. (A) Specificity of *KCNJ11* qMSP assay for the methylated primers set: amplification curves of 100%, 50%, 10%, 5% and 0.5% SB-converted non-methylated DNA standards. (B) Specificity of *IAPP* qMSP assay for the unmethylated primers set: amplification curves of 100%, 50%, 10%, 5% and 0.5% SB-converted non-methylated DNA standards. (C) Sensitivity of *GCK* qMSP assay for the unmethylated primers set: amplification curves of 10-fold serially diluted 100% SB-converted non-methylated DNA standard—efficiency 98%. (D) Sensitivity of *IAPP* qMSP assay for the methylated primers set: amplification curves of 10-fold serially diluted 100% SB-converted non-methylated DNA standard—efficiency 91%.
